# Big Data and Artificial Intelligence for Precision Medicine in the Neuro-ICU: Bla, Bla, Bla

**DOI:** 10.1007/s12028-021-01427-6

**Published:** 2022-01-12

**Authors:** Giuseppe Citerio

**Affiliations:** 1 Department of Medicine and Surgery, University of Milano-Bicocca, Monza, Italy; 2 Neuro-Intensive Care Unit, Neuroscience Department, San Gerardo Hospital, Monza, Italy

**Keywords:** Artificial intelligence, Big data, Neurointensive care

“Bla, bla, bla.” With these words, Greta Thunberg condemned the world leaders in Milan over the climate crisis. She said there are many fine words, but the science does not lie: CO_2_ emissions are still rising. The same could apply to artificial intelligence (AI) in neurocritical care. There are plenty of articles, conference talks, a lot of bla, bla, bla but no real AI application changing our approach in managing neurocritical care patients. The AI inventers in the previous century had envisioned a future with technologies that could sense and think like, or even better than, clinicians at the bedside, a hypothesis that is probable to linger in the territories of science fiction in my remaining professional career.

Please, do not misunderstand me. I envisage a future neurocritical care system with AI that can phenotype the patient, anticipate any pathophysiological derangements early, evaluate the better strategy to be applied in the individual neurocritical care patients, suggest it to the clinician in charge, and evaluate the response to the intervention, learning from the response of the patients. I would love to have such systems working properly, guiding and supporting me and my colleagues, but the reality is that we are barely there yet, and I do not see a strong, concrete, coordinate movement for reaching this aim.

Today, only used for quoting some potential scenarios in neurocritical care, AI is not guiding intracranial hypertension therapies at the bedside, seizure or stroke treatments, or disorder of consciousness understanding.

The intracranial pressure management strategies are summarized in a simple stepwise approach for everyone [1, 2]. The effect of the ICP dose [3–5] has been explored, but it has not yet been integrated in clinical reasoning. The selection of strategies remains not individualized and without any AI-guided approach [6].

Some advancements in seizure detection have been trumpeted with AI using data from conventional electroencephalography (EEG). Reasonable sensitivity and specificity were described [7, 8], but it did not become routine practice. These findings are limited, and generalizability is still an issue.

Some tools available today are US Food and Drug Administration approved [9], such as the Rapid software for the detection of large cerebral vessel occlusion (LVO). It identifies suspected LVO with a high sensitivity and specificity in a couple of minutes and notifies the stroke team members when suspected LVO is detected. This is great but it is not enough [10]. We are looking for a clinical decision support tool that, when combining anamnestic, clinical, and imaging information, generates accurate estimations of likely outcomes under different treatment scenarios and suggests the best one and then provides an evaluation of the results that will be integrated for ameliorating the future algorithm.

Evidence of covert consciousness in 15% of individuals with absence of behavioral responses to motor commands following an acute brain injury was confirmed by supervised learning algorithms from EEG [11]. Moreover, 12 h following cardiac arrest, a deep-learning artificial neural network trained on EEG data in comatose patients was able to predict 6-month functional outcome [12].

In my opinion these advances are interesting but minimal, and the potential of AI in neurocritical care has still to blossom. The so-called AI chasm [13], the hiatus between developing “a scientifically sound algorithm and its use in any meaningful real-world applications,” might explain the situation we are experiencing. The AI experts focus their efforts on the technical aspects of the algorithms, with scarce consideration for the interaction with human users, in our setting, the neurointensivists. Because neurointensivists and neurointensive care unit nurses play the pivotal role in patient care, the development and the evaluation of AI-based clinical algorithms have to be focused on augmenting rather than replacing human intelligence. However, AI-based decision support systems frequently lack the possibility to be understood in their logic (the so-called black box problem) and threaten the historical medical decision-making process. Hence, the challenge is to keep neurointensivists at the center of the design and assessment route of the new AI applications.

A potential solution to the AI chasm is an early limited clinical evaluation step between the in silico development of the algorithm and a large-scale clinical trial for evaluating AI applications.

Clinical decision-making processes are complicated, and we cannot expect that human users, i.e., the neurointensivists, who remain responsible for their clinical decisions, will exactly obey all the recommendations from an algorithm with perceived opacity as an inscrutable black box [14, 15]. Therefore, it is essential to assess the actual assisted human performance and algorithm usability at an early stage in the target patient population to confirm its relevance in the implementation settings and the need to be reported as a crucial outcome. Moreover, we are not sure that clinicians’ decisions will mirror the AI recommendations. We need to test the safety profile of new algorithms when used to guide human decisions to avoid exposing to risk of harm a large group of critically ill patients.

AI ergonomics should occur as early as possible, and it requires reiterative evaluation design cycles. Technical necessities often progress as a system starts being employed, and clinicians’ expectations of the performance of an AI system also grow after the initial acquaintance period, requiring, for example, additional variables to improve the algorithm’s recommendations. This would demand AI creators to integrate other parts of the electronic patient record.

These iterative design modifications and rapid prototyping are essential and have to be planned early and not during large-scale trials to avoid undermining the whole project. This step might be compared to a phase 1 or 2 trial for drug development. However, before using the new AI tool at the bedside, we need to have a large trial demonstrating an efficacy on valuable outcomes, as we are used to with phase 3 trials before adopting a new therapeutical approach. However, this stage cannot be escaped. Large-scale clinical trials are complicated and expensive activities that require meticulous preparation. A well-planned design is fundamental for obtaining acceptable and meaningful conclusions, and it requires background information about the therapeutical strategy under evaluation. The silico evaluation cannot give all this information, and some has to be gathered in limited prospective studies. Some fundamental information has to be defined while trial protocols are drafted, for example, the most suitable outcomes for the trial, the expected effect size, the best inclusion and exclusion criteria, the evolution of the users’ confidence in the algorithm, and how to use the algorithm’s output. This path toward an integration of AI in the care of the sickest neurointensive care patients is a long route that needs a collaborative effort and funding and, as highlighted in a recent World Health Organization publication [16], requires the satisfaction of some core principles to promote its ethical use: protect autonomy; promote human well-being, human safety, and the public interest; ensure transparency, explicability and intelligibility; foster responsibility and accountability; ensure inclusiveness and equity; and promote AI that is responsive and sustainable.

Topol [17] wrote the following:

The greatest opportunity offered by AI is not reducing errors or workloads, or even curing cancer: it is the opportunity to restore the precious and time-honored connection and trust—the human touch—between patients and doctors. Not only would we have more time to come together, enabling far deeper communication and compassion, but also we would be able to revamp how we select and train doctors.

I hope we will stop the bla, bla, bla and start to build a better plan for implementing AI in medicine and in neurocritical care using the opportunity that technology will offer us to be better doctors at the bedside.
